# Prevalence of Local Immune Response against Oral Infection in a *Drosophila*/*Pseudomonas* Infection Model

**DOI:** 10.1371/journal.ppat.0020056

**Published:** 2006-06-09

**Authors:** Peter Liehl, Mark Blight, Nicolas Vodovar, Frédéric Boccard, Bruno Lemaitre

**Affiliations:** Centre de Génétique Moléculaire, Centre National de la Rercheche Scientifique, Gif-sur-Yvette, France; Stanford University, United States of America

## Abstract

Pathogens have developed multiple strategies that allow them to exploit host resources and resist the immune response. To study how *Drosophila* flies deal with infectious diseases in a natural context, we investigated the interactions between *Drosophila* and a newly identified entomopathogen, Pseudomonas entomophila. Flies orally infected with P. entomophila rapidly succumb despite the induction of both local and systemic immune responses, indicating that this bacterium has developed specific strategies to escape the fly immune response. Using a combined genetic approach on both host and pathogen, we showed that P. entomophila virulence is multi-factorial with a clear differentiation between factors that trigger the immune response and those that promote pathogenicity. We demonstrate that AprA, an abundant secreted metalloprotease produced by *P. entomophila,* is an important virulence factor. Inactivation of *aprA* attenuated both the capacity to persist in the host and pathogenicity. Interestingly, *aprA* mutants were able to survive to wild-type levels in immune-deficient *Relish* flies, indicating that the protease plays an important role in protection against the *Drosophila* immune response. Our study also reveals that the major contribution to the fly defense against P. entomophila is provided by the local, rather than the systemic immune response. More precisely, our data points to an important role for the antimicrobial peptide Diptericin against orally infectious Gram-negative bacteria, emphasizing the critical role of local antimicrobial peptide expression against food-borne pathogens.

## Synopsis

Normal feeding and digestion involves the ingestion of many microorganisms. Many are innocuous, some are commensal, and others may be pathogenic. Eukaryotes have thus evolved complex mechanisms to detect, control, and if necessary, eliminate intestinal microbes. Insects are no exception, and the fruit fly, *Drosophila,* employs a physical barrier within the intestinal lumen and the peritrophic matrix, and an innate immune response which exhibits similarities to the mammalian counterpart. Pseudomonas entomophila was identified as a novel entomopathogenic bacterium that can infect and colonize the gut of *Drosophila.* In this paper, Liehl et al. describe one specific secreted virulence factor of *P. entomophila,* the zinc metalloprotease, AprA, which they demonstrate to be required for defense against the host gut epithelial immune response. AprA defends P. entomophila against the *Drosophila* antimicrobial peptides, produced by the gut innate immune response. *P. entomophila aprA* mutants are attenuated for virulence in wild-type *Drosophila* but are equally infective as wild-type bacteria in immune-deficient mutant flies that do not express these antimicrobial peptides. Although secreted proteases have previously been described as a potentially important defense against host immune proteins, this is one of the rare examples of an in vivo demonstration of such a specific role against insect antimicrobial peptides.

## Introduction

Host-pathogen interactions are complex relationships in which the success of each organism depends on its ability to overcome the other. Consequently, hosts have evolved surveillance and defense mechanisms to detect and eliminate invading microorganisms, whereas pathogens use sophisticated strategies to counteract such responses. In recent years, *Drosophila* has emerged as a powerful model for the study of host-pathogen interactions [1,2]. An attractive feature of the *Drosophila* system is the existence of multiple defense reactions that are shared with higher organisms [3–5]. These strategies include physical barriers, together with the local and systemic immune responses. Several tissues participate in a coordinated defense against microbial infection. Firstly, epithelia, such as alimentary tract and tracheae, are the first line of defense against pathogens and produce both antimicrobial peptides (AMPs) and reactive oxygen species. Secondly, specialized hemocytes participate in phagocytosis and encapsulation of foreign intruders. Finally, the fat body, a functional equivalent of the mammalian liver, is the site of the humoral response. Several genetic studies have demonstrated the regulation of AMP synthesis by the Toll and immune-deficient (Imd) pathways. The Toll pathway is mainly activated by Gram-positive bacteria and fungi, and controls, to a large extent, the expression of AMPs (e.g., *Drosomycin*) through the nuclear factor-κB transactivators Dif and Dorsal. In contrast, the Imd pathway mainly responds to Gram-negative bacterial infections and controls different AMP genes (e.g., *Diptericin*) via the activation of the nuclear factor-κB transactivator Relish (Rel) [3,4,6]. In addition, the Imd pathway plays a predominant role in the regulation of AMPs in the alimentary tract and tracheal epithelia [7].

Our knowledge of the *Drosophila* immune response is mainly based on the analysis of host reactions following direct injection of non-pathogenic bacteria into the insect hemocoel. One limitation associated with this approach is that it bypasses the initial steps of naturally occurring infections, including bacterial colonization and persistence and host local immune responses. Septic injury is probably of little consequence in nature, unlike oral infection upon ingestion. In addition, the response to non-pathogenic microorganisms does not necessarily reflect a natural host reaction to a real pathogen. Recently we have developed the use of natural oral infection to dissect host responses of *Drosophila* after challenge with bacteria demonstrated to be infectious for this insect. We have isolated several Erwinia carotovora strains, such as *Ecc15,* for their capacity to persist in the *Drosophila* larval gut and to trigger a strong systemic immune response following oral infection [8]. Although not a pathogen to *Drosophila,* use of *Ecc15* has been pivotal in revealing the ability of *Drosophila* to activate an adapted response to persistent microorganisms in the gut, including the induction of local immune responses. More recently, we isolated *Pseudomonas entomophila,* a Gram-negative natural bacterial pathogen of *Drosophila,* from flies in Guadeloupe [9]. We demonstrated that P. entomophila induces both systemic and local immune responses in *Drosophila* and causes a food-uptake cessation following oral infection in larvae. Importantly, in contrast to *Ecc15, P. entomophila* is highly pathogenic for *Drosophila* as well as for species from a different insect order *(Lepidoptera).* The genome sequence of P. entomophila has been completed and reveals the existence of a large set of genes encoding putative virulence factors such as proteases, lipases, toxins, alginate synthesis, and adhesion factors [10]. Transposon mutagenesis of P. entomophila allowed us to identify several regulatory genes required to infect or to kill *Drosophila*. Among them we identified the GacS/GacA two-component regulatory system which, in *Pseudomonas fluorescens,* has been well characterized and demonstrated to be an essential regulator of virulence [11]. *P. entomophila gacA* mutants are non-pathogenic and fail to trigger a systemic immune response after ingestion, indicating that the GacS/GacA two-component system regulates important bacterial virulence effectors required to infect and kill flies [9].

Despite these studies, little is known about the mechanisms used by bacterial pathogens to interfere with the corresponding insect host defenses. Here we report an in vivo analysis of P. entomophila virulence to both *Drosophila* larvae and adult flies following natural oral infection. We present in vivo evidence for a direct role in pathogenesis of the secreted P. entomophila zinc metalloprotease, AprA, which counteracts AMPs synthesized by the host. Furthermore, we demonstrate that the *Drosophila* Imd-regulated local gut response is necessary to combat infection and that P. entomophila AprA-regulation via GacS/GacA and PrtR regulators is used as a strategy to escape AMP activity. This study also highlights the importance of the gut immune response in the control of food-borne pathogens.

## Results

### P. entomophila Secretes an Abundant Protease

**Figure 1:**
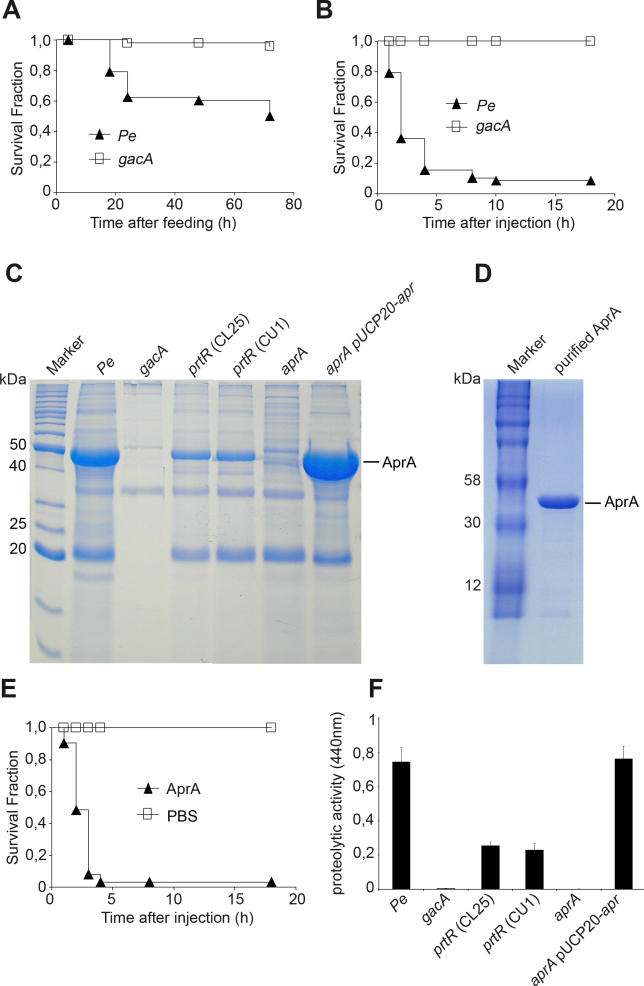
Identification and Regulation of a P. entomophila Protease (A) Survival analysis of wild-type (Or^R^) larvae following feeding with 60-fold concentrated sterile filtered culture supernatants of wild-type *P. entomophila* and its *gacA* mutant. Survival analysis was repeated three times and performed on 50 larvae. Log-rank analysis demonstrated a statistically significant difference in survival of flies fed with supernatant from wild-type P. entomophila and flies fed with supernatant from the *gacA* mutant (*p* < 0.0001). (B) Survival analysis of wild-type (Or^R^) adult flies (*n* = 60) injected with 69 nl of non-concentrated supernatants of wild-type P. entomophila and its *gacA* mutant. Log-rank analysis demonstrated a statistically significant difference in survival of flies injected with supernatant from wild-type P. entomophila and flies injected with supernatant from the *gacA* mutant (*p* < 0.0001). (C) SDS-PAGE analysis of culture supernatants from P. entomophila derivatives. Protein extracts from culture supernatants (OD_600_ = 2) following growth at 29 °C for 24 h, were loaded and stained with Coomassie blue. The genotypes of the bacterial strains used are indicated on the top. The first lane represents the molecular weight marker (indicated in kDa on the left). CL25 and CU1 are two independent Tn*5* insertions in the *prtR* locus [10].The last lane displays the *aprA* mutant complemented with a plasmid expressing the *aprA* locus. AprA corresponds to the major band at 51 kDa. (D) Purification of P. entomophila AprA. Lane 1, molecular weight marker. Lane 2, purified peak fraction of AprA. (E) Survival analysis of wild-type adult flies (*n* = 60) following microinjection of 9.2 nl of purified AprA protease (50 μg/ml) or PBS. Flies succumbed within 4 hr after microinjection of pure protease. Log-rank analysis demonstrated a statistically significant difference in survival of flies injected with AprA and flies injected with PBS (*p* < 0.0001). (F) Proteolytic activity of supernatant of P. entomophila derivatives as measured at 440 nm by the azocasein test.

A common strategy used by bacterial pathogens is to secrete toxins and other virulence factors that damage host tissues. To test whether P. entomophila could secrete such toxic factors, a supernatant filtrate from a bacterial culture was tested for its ability to kill *Drosophila.* Figure 1A shows that a concentrated P. entomophila culture filtrate had a moderate but significant effect on *Drosophila* larvae survival following ingestion. Although the filtrate did not kill adult flies after feeding (unpublished data), it was highly toxic by direct injection into the hemocoel (Figure 1B). No killing of either larvae or adults was observed with a culture filtrate derived from the avirulent P. entomophila strain carrying a Tn*5* transposon in the *gacA* gene [9]. These data suggested that P. entomophila secreted one or more factors with toxic activity under the control of the GacS/GacA two-component system.

In an attempt to identify the factor(s) responsible for toxicity, we analyzed the proteins present in the supernatant. The protein profile for wild-type P. entomophila supernantant (Figure 1C, lane 2) shows a major protein band at 51 kDa and several minor bands. MALDI-TOF analysis of tryptic fragments of the 51-kDa band identified this protein as a homolog of the previously characterized Apr proteases from Pseudomonas spp., Prt proteases from Erwinia spp. and Photorhabdus spp., and serralysins from Serratia spp. [12–14]. All of these proteases are members of the zinc metzincin family of Type I-secreted RTX proteins [15]. We subsequently purified this protease, termed AprA, to homogeneity from the P. entomophila supernatant by anion exchange chromatography followed by size exclusion chromatography (Figure 1D). Injection of pure AprA into the hemocoel rapidly killed adult flies, identifying this protein as a bacterial toxin (Figure 1E). Feeding of larvae with high concentrations (1.5 mg/ml) of AprA led to modest lethality (unpublished data), recapitulating the properties of the P. entomophila supernatant.

Interestingly, in a *gacA* mutant the number of proteins in the supernatant was greatly reduced, including the complete absence of the 51-kDa band corresponding to AprA (Figure 1C, lane 3). In *P. fluorescens,* membrane-localized anti-sigma factor PrtR cleaves the extracytoplasmic sigma factor PrtI, resulting in increased expression of multiple genes, including one encoding for a metalloprotease, *aprX* [16]. Two independent Tn*5* insertions in the *prtR* gene affecting virulence have recently been identified in a random insertional mutagenesis screen of P. entomophila [10]. To test a possible role of *prtR* in the regulation of *aprA* expression, we analyzed the proteins present in the supernatant of *prtR* mutants. Interestingly, both *prtR* mutants (CL25 and CU1) displayed a secreted protein profile identical to the wild-type strain except for a marked decrease of AprA (Figure 1C, lanes 4 and 5). Measurement of in vitro protease activity with azocasein revealed that the *gacA* mutant secretes no detectable protease activity and that *prtR* mutants retain only 30% of wild-type supernatant activity, thus indicating a role for PrtR in the regulation of P. entomophila protease secretion (Figure 1F). Furthermore, the *prtR* supernatant showed no toxicity toward flies after injection, suggesting a correlation between AprA levels and virulence (unpublished data). Altogether, this analysis indicates that *P. entomophila gacA* and *prtR* genes regulate the secretion of a protease with toxic activity when injected into flies or fed to larvae.

### The *P. entomophila aprA* Mutant Displays Attenuated Pathogenicity

**Figure 2:**
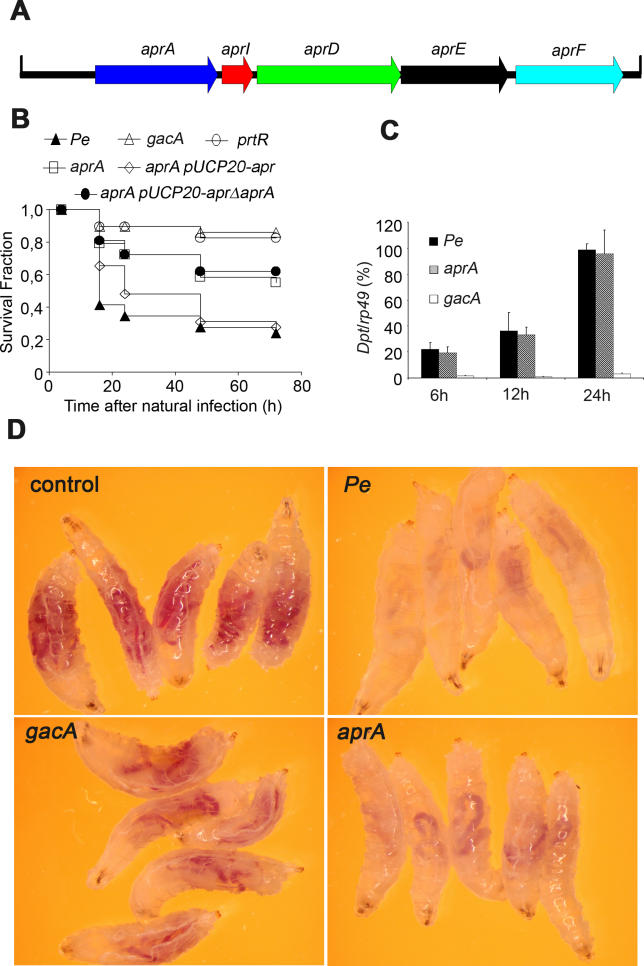
The *aprA* Mutant Exhibits Attenuated Virulence (A) Genetic organization of the *P. entomophila apr* locus and its associated Type 1 transporter. The locus contains the structural gene for the protease *(aprA),* followed by the genes encoding its putative inhibitor *(aprI)* and those coding for the associated Type 1 transporter (ABC Transporter, *aprD;* Membrane Fusion Protein*, aprE;* Outer Membrane Protein*, aprF*). The *apr* operon organization was deduced from [10]. (B) Survival analysis of *Drosophila* larvae (*n* = 60) after feeding with wild-type *P. entomophila, gacA, prtR,* and *aprA* mutants; the *aprA* mutant complemented with the wild-type *apr* operon *(pUCP20-apr);* and an *aprA* mutant complemented with the *apr* operon carrying a non-polar mutation in the *aprA* gene *(pUCP20-apr*Δ*aprA).* This experiment was repeated twice and yielded similar results. Log-rank analysis demonstrated a statistically significant difference in survival of flies fed with wild-type P. entomophila and flies fed with the *aprA* mutant (*p* < 0.0001). (C) *Diptericin* expression measured by RT-qPCR in *Drosophila* larvae following natural infection with wild-type *P. entomophila, aprA,* and *gacA* mutants. Infection with wild-type P. entomophila and the *aprA* mutant induced sustained *Diptericin* expression unlike the *gacA* mutant. Larvae were collected at different time intervals after oral infection. *Diptericin* expression was normalized to *rp49* mRNA. For each time point the values represented are the mean of three independent experiments (± standard deviation). 100% value corresponds to the level of *Dpt* mRNA obtained 24 h after infection with wild-type P. entomophila. *rp49*: *ribosomal protein 49*. (D) Ingestion of *aprA* or *prtR* mutant bacteria induces a food-uptake cessation in larvae in contrast to animals fed with the *gacA* mutant. Larvae fed with medium containing 0.5% (w/v) bromophenol blue displayed a clearly discernable blue coloration throughout the gut whereas larvae fed with both P. entomophila wild-type, *aprA,* or *prtR* mutants and bromophenol blue showed only a pale blue coloration. Images were taken 6 h after infection. This visual effect was not due to a change of gut pH (acidification) that would result in yellow rather than blue coloration since the overall level of bromophenol blue in dissected and homogenized intestines was confirmed by measuring the absorbance of blue dye with larval extracts in a HEPES Buffer at [pH 8] (unpublished data).

The experiments described above suggested that AprA plays an important in vivo role in P. entomophila virulence. To further test this hypothesis, we used a genetic approach by inactivating the *aprA* gene. Sequence analysis of the locus corresponding to *aprA* revealed a genetic organization characteristic of this class of Type I-secreted proteases [14]. The structural gene for the metalloprotease, *aprA,* is followed by a gene encoding its periplasmic inhibitor, *aprI,* and three genes encoding the Type I transporter, *aprD, E,* and *F* (Figure 2A). Studies in other bacteria have shown that AprD, E, and F participate in the elaboration of a Type I transporter required for AprA secretion to the external medium [17]. We inactivated the *aprA* gene by inserting a tetracycline resistance cassette by a double homologous recombination event. SDS-PAGE protein profiles of culture supernatants clearly demonstrated the absence of the 51-kDa band, and in vitro protease activity of the *aprA* mutant confirmed the concomitant absence of secreted protease activity (Figure 1C, lane 6, and 1F).

**Figure 3:**
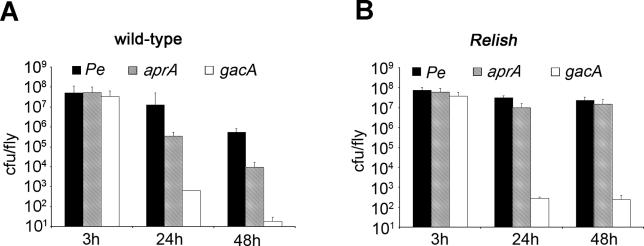
Bacterial Persistence in Wild-Type and *Rel* Flies (A) Bacterial persistence was measured in live wild-type flies by plating appropriate dilutions of homogenates of five surface-sterilized adults on LB medium containing rifampicin. The flies had been previously orally infected with *rif^R^* strains of wild-type P. entomophila and its *aprA* and *gacA* derivatives. *AprA* mutants persisted less than wild-type P. entomophila but better than the *gacA* mutant. (B) Persistence of wild-type *P. entomophila aprA* and *gacA* mutants in live *Rel* flies. *AprA* mutant bacteria persisted at a level similar to wild-type bacteria in *Rel* mutant flies whereas *gacA* bacterial levels decreased with time. The number of cfus per fly represented in each histogram corresponds to the average of six independent experiments (± standard deviation)*. cfu, colony-forming unit.*

To analyze the in vivo contribution of AprA to P. entomophila virulence, we performed a survival analysis of *Drosophila* larvae and adults after oral infection with various P. entomophila derivatives. Only 40% of the *aprA-*infected larvae succumbed within 3 days, while 70% of the larvae died after infection with wild-type *P. entomophila,* demonstrating that the *aprA* mutant was attenuated for virulence (Figure 2B). Similarly, the *aprA* mutant showed reduced pathogenicity after oral infection of adult flies (Figure S1). Using the same assay, both *prtR* and *gacA* mutants were almost avirulent toward *Drosophila* indicating that both genes regulate other virulence factors in addition to AprA (Figure 2B). We next compared persistence of wild-type P. entomophila and the *aprA* mutant by quantifying the number of bacteria in larvae and adults at different time points after infection. Whereas the wild-type P. entomophila titer remained high, *aprA* bacterial levels decreased significantly with time (Figure 3A, not shown for larvae). However, *aprA* gut persistence remained higher than that of *gacA* mutants.

We could not exclude that the *aprA* mutant phenotype was due to polar effects of the insertion in the operon upon expression of *apr I, D, E,* and *F.* In support of this hypothesis, previous studies have demonstrated for both Serratia marcescens and P. fluorescens that Type I transporters can be polyvalent and secrete different types of proteins such as proteases and lipases [18,19]. Complementation of the *aprA* mutant with the wild-type *apr* operon restored protease secretion and activity (Figure 1C, lane 7, and 1F) and resulted in a complete regain of function with respect to all virulence phenotypes (Figures 2B and S1). In contrast, no rescue was observed with a plasmid containing the full *apr* operon but carrying a non-polar mutation in *aprA* (Figures 2B and S1). These data confirmed that the protease AprA itself contributes to P. entomophila virulence.

### The *aprA* Mutant Retains the Capacity to Trigger an Immune Response and to Induce Food-Uptake Cessation

In addition to its pathogenicity, P. entomophila infection provokes a food-uptake cessation in larvae and triggers an immune response in both larvae and adults [9]. Feeding of larvae with medium containing bromophenol blue, leads to a clearly visible food uptake, discernable by blue coloration throughout the gut. In contrast, P. entomophila-infected larvae show only a pale blue coloration indicating that infected larvae cease to ingest food. Whereas this food-uptake cessation was still observed in *aprA* and *prtR* mutants, it was not evident in *gacA* mutant-infected larvae (Figure 2D and data not shown for *prtR*).

The kinetics of the expression of the AMP, *Diptericin,* in larvae and adults after natural infection by P. entomophila and its derivative mutants, was analyzed by real time quantitative PCR (RT-qPCR). *Diptericin* was expressed at a wild-type level following oral infection with the *aprA* and *prtR* mutants, but no expression was detected in the case of the *gacA* mutant (Figures 2C and 5A). Observation of green fluorescent protein (GFP) fluorescence in infected larvae carrying a *Diptericin-GFP* reporter gene confirmed that *prtR* and *aprA* mutants elicited an immune response, whereas *gacA* mutants failed to do so (unpublished data).

The data presented above clearly indicate that AprA is a major virulence factor of P. entomophila that promotes bacterial persistence and killing of the host, but that other virulence factors controlled by *gacA* and *prtR* exist. It also reveals that P. entomophila virulence is multi-factorial with a clear distinction between factors that promote pathogenicity and those that trigger the immune response. The observation that both factors are under the control of the GacS/GacA, confirmed that this two-component system is the master regulator of P. entomophila virulence.

### AprA Confers Protection against the Imd-Dependent Immune Response

Virulence factors allow pathogens to survive in the hostile environment of the host, to escape the immune system, or to establish a biotope where they can proliferate by altering host functions. The observation that P. entomophila persistence in flies and larvae is lower in *aprA* mutants clearly indicates that the AprA protease promotes bacterial persistence in the *Drosophila* gut. Our previous study had already revealed that the humoral arm of the *Drosophila* immune response mediated by the Imd pathway contributes to resistance against orally transmitted *P. entomophila,* since flies mutated in the *Relish (Rel)* gene showed a consistently increased mortality rate [9]. To determine whether AprA plays a role in counteracting the Imd-dependent immune response, we compared persistence of wild-type *P. entomophila, gacA,* and *aprA* derivatives by quantifying the number of bacteria in *Rel* flies and larvae. Whereas the titer of *gacA* mutants decreased with time, we observed that the *aprA* derivative persisted at a level similar to wild-type bacteria in *Rel-*deficient mutant flies and larvae (Figure 3A and 3B, and unpublished data). The observation that AprA was required to promote persistence in wild-type flies and larvae but was dispensable in a *Rel* background, indicates that AprA plays an important role in the protection against the Imd-dependent immune response.

**Figure 4:**
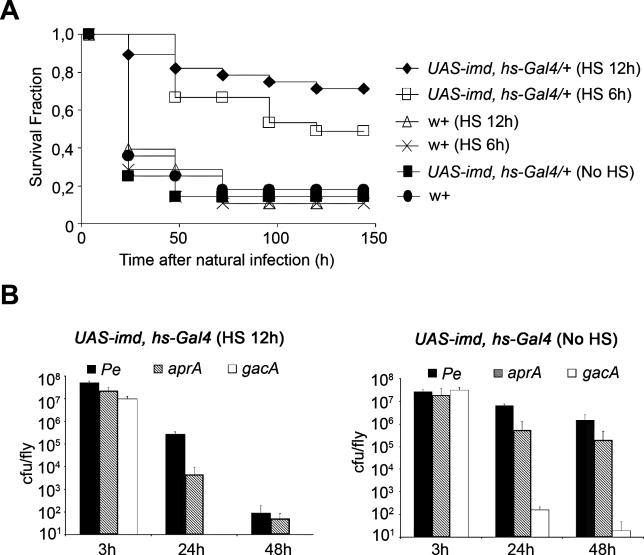
AprA Confers Protection against the Imd-Dependent Immune Response (A) Over-expression of the Imd immune pathway protects against P. entomophila infection during gut infection. The genotypes of the flies are as indicated. Over-expression of the *UAS-imd* construct with an *hs-GAL4* driver induces strong expression of the *Diptericin* gene in the absence of infection [39]. The expression was triggered once (1 h heat-shock at 37 °C) 6 h and 12 h prior to infection. The percentage of surviving flies (*n* = 60) after infection with wild-type P. entomophila is shown. This experiment was repeated three times and gave similar results. Log-rank analysis demonstrated a statistically significant difference in survival of wild-type flies and *UAS-imd, hs-Gal4/+* (HS 12 h) flies fed with wild-type P. entomophila (*p* < 0.0001). HS, heat-shock. (B) Persistence of *aprA, gacA,* and wild-type P. entomophila in adults over-expressing *imd* 12 h prior to infection. *AprA* bacterial titers decrease faster with time than the ones of wild-type bacteria. The *gacA* mutant fails to survive in the intestine of flies over-expressing *imd*. The number of cfus per fly represented in each histogram corresponds to the average of six independent experiments (± standard deviation). cfu, colony-forming unit.

To investigate the connection between activation of the Imd pathway and the outcome of P. entomophila infection in more detail, we focused our analysis on the adult stage when it is easier to monitor survival. P. entomophila infection triggers Imd pathway activation within hours. To determine at which time Imd was required to impede P. entomophila infection, we monitored survival to P. entomophila in flies in which the Imd pathway was artificially activated in a time-dependent manner. We utilized an engineered fly line *(UAS-imd, hs-Gal4)* over-expressing *imd* under the control of a heat-shock promoter via the UAS-Gal4 system, which consequently leads to strong expression of Imd-dependent AMP genes. Figure 4A shows that over-expressing *imd* 12 h prior to infection protected flies from a P. entomophila infection, whereas over-expressing *imd* 6 h before contributed only modestly to survival of the flies. This experiment indicates that P. entomophila can be eliminated when the Imd pathway is activated at a high level prior to infection. It further suggests that P. entomophila is sensitive to immune defenses during a short time frame and subsequently becomes insensitive. Since P. entomophila is pathogenic under normal wild-type conditions, it may overcome the immune response by establishing a niche in the gut before the Imd pathway is activated. It is interesting to note that in agreement with the results described above, persistence of both the *aprA* mutant and wild-type bacteria was significantly altered in fly lines over-expressing the Imd pathway, and that *aprA* mutants survived less than wild-type bacteria (Figure 4B). Altogether, these data reveal that the Imd pathway is crucial for host survival after oral infection with P. entomophila and that AprA plays a key role in counteracting this effect.

### Local, but Not Systemic Immunity, Contributes to Resistance against Oral Infection with P. entomophila

**Figure 5:**
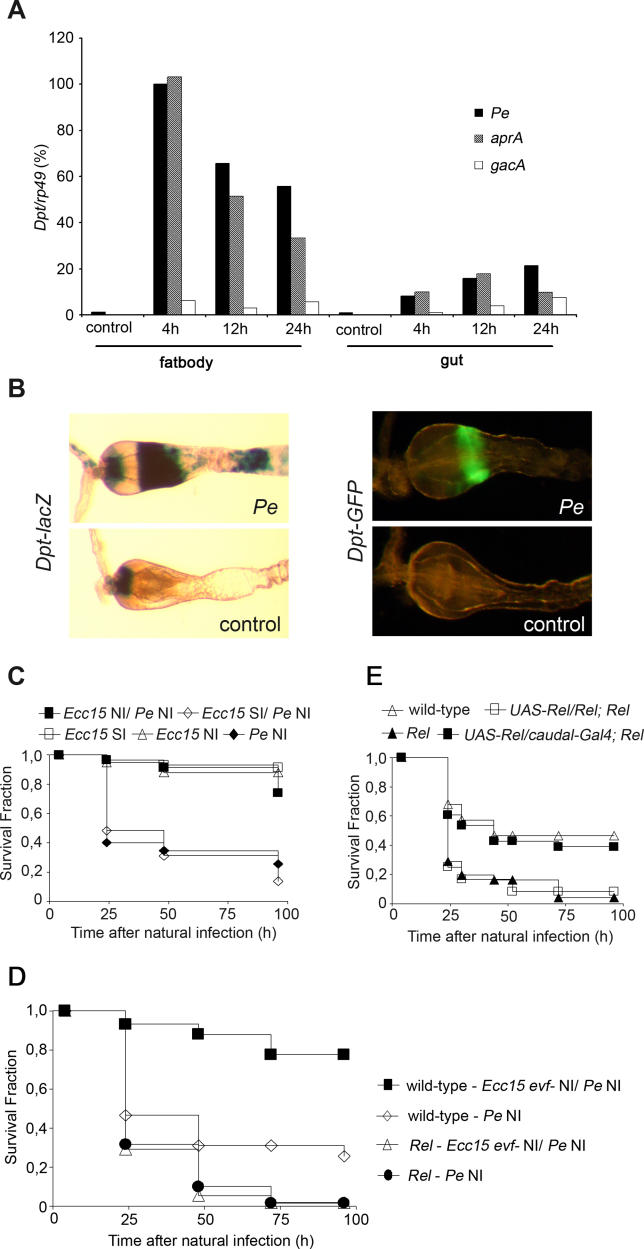
The Local Immune Response Plays a Critical Role against P. entomophila (A) Time course analysis of *Diptericin* expression measured by RT-qPCR in *Drosophila* fat body and gut extracted from females following natural infection with wild-type *P. entomophila, aprA,* and *gacA* mutants. Infection with wild-type P. entomophila and the *aprA* mutant induced a rapid and sustained *Diptericin* expression unlike the *gacA* mutant. Fat bodies (carcasses) and digestive tracts were dissected from adults collected at different time intervals after oral infection. *Diptericin* expression was normalized to *rp49* mRNA. 100% value corresponds to the level of *Dpt* mRNA obtained 4 h after infection with wild-type P. entomophila. *rp49*: *ribosomal protein 49*; *Dpt, Diptericin* (B) *P. entomophila* induces *Diptericin* expression in the cardia of adult *Drosophila*. Histochemical staining of β-galactosidase activity shows that *Dpt-lacZ* is expressed in the anterior midgut at the level of the proventriculus of infected flies carrying the *Dpt-lacZ* reporter gene (left panel). Similar results were obtained with a *Dpt-GFP* transgene (right panel)*.* Adults were collected 24 h after infection. The control pictures display the cardia of uninfected flies. A constitutive expression was observed in the anterior part of the cardia. *Dpt, Diptericin*. (C) Survival of wild-type flies (*n* = 60) to P. entomophila after previous infection with *Ecc15*. Flies were first infected with *Ecc15* either orally (OD_600_ = 100) or by septic injury (OD_600_ = 200). 20 h after this *Ecc15* infection, flies were fed with P. entomophila. Survival curves demonstrate that *Drosophila* flies primed with *Ecc15* were protected from a subsequent P. entomophila infection only when *Ecc15* was orally administrated. Log-rank analysis demonstrated a statistically significant difference in survival to P. entomophila infection of flies primed with *Ecc15* by septic injury (SI) or natural infection (NI) (*p* < 0.0001). (D) Survival of wild-type and *Rel* flies (*n* = 60) to P. entomophila after previous infection with *Ecc15 evf-*. Flies were first infected with *Ecc15 evf-* orally (OD_600_ = 100). 20 h after this *Ecc15* infection, flies were fed with P. entomophila. Survival curves demonstrate that wild-type, but not *Rel Drosophila* flies primed with *Ecc15 evf-*, were protected from a subsequent P. entomophila infection. This experiment was repeated three times and gave similar results. Log-rank analysis demonstrated a statistically significant difference in survival to P. entomophila infection of flies primed with *Ecc15 evf-* by natural infection (NI) and flies without previous priming (*p* < 0.0001). (E) *Rel* flies expressing a *UAS-Rel* transgene in the midgut under the control of the gut-specific *cad-Gal4* driver survive better to P. entomophila infection than *Rel* mutant flies. Survival experiments were performed on 30 flies orally infected with P. entomophila (OD_600_ = 50). This experiment was repeated three times and yielded similar results. Log-rank analysis demonstrated a statistically significant difference in survival of *UAS-Rel/caudal-Gal4; Rel* flies, and *UAS-Rel/Rel; Rel* flies to P. entomophila infection (*p* < 0.01).

*P. entomophila,* but not the *gacA* derivative, rapidly activates both the local and systemic Imd-dependent immune responses in *Drosophila* adults, which can be demonstrated using RT-qPCR with fat body and gut extracts (Figure 5A). Use of *Diptericin-GFP* or *lacZ* reporter genes reveals strong *Diptericin* expression in the proventriculus, an organ that acts as a valve between the oesophagus and the anterior midgut (Figure 5B). This suggests a critical role of this organ in the elimination of bacteria. We next examined the respective contribution of the local gut and the systemic immune responses to controlling P. entomophila infection. We thus compared resistance to P. entomophila infection of flies that were previously either orally infected with *Ecc15* (to activate a local immune response) or pricked with *Ecc15* (to activate a systemic immune response). The rationale behind this experiment is based on previous observations that an oral infection with *Ecc15* triggers a local, but not a systemic immune response at the adult stage (our unpublished data) [8]. Figure 5C shows that a prior infection with *Ecc15* protected flies against a subsequent P. entomophila infection only when *Ecc15* was administrated orally, indicating that the local, but not the systemic immune response, plays an important role in P. entomophila clearance. To ascertain that the protection was due to Imd pathway activation and not to a competition between P. entomophila and *Ecc15,* we next monitored resistance to P. entomophila of wild-type and *Rel* flies orally infected by *Ecc15 evf-*. We used the *evf-*deficient derivative because this bacterium triggers a local immune response without persisting in the gut. This experiment shows that wild-type, but not Rel flies previously infected with *Ecc15 evf-,* resisted a second challenge by P. entomophila (Figure 5D). This demonstrates that the protection was indeed due to Imd pathway activation in the gut.

The results described above were corroborated by the over-expression of Imd in the gut or in the fat body using specific Gal4 drivers. Over-expression of Imd in the gut protected against *P. entomophila,* whereas its activation in the fat body did not (Figure S2). However, the previous results did not address whether the Imd pathway induced during the course of a natural P. entomophila infection contributes to fly resistance to *P. entomophila.* Thus, we next compared survival of *Rel* flies specifically expressing a wild-type copy of *Rel* in the intestine using *caudal (cad)-Gal4*. These flies lack a functional Imd pathway except in the gut where *cad-Gal4* is expressed. In these flies, the Imd pathway was not constitutively active in the gut but could be induced upon oral bacterial infection similarly to the wild-type situation (Figure S3A). Figure 5E shows that *Rel* flies expressing *Rel* in the gut survive better than *Rel* mutant flies. These data demonstrate that the P. entomophila-induced local immune response in the intestine plays an important role in the defense against this Gram-negative bacterium.

### AprA Confers Protection against Diptericin

**Figure 6:**
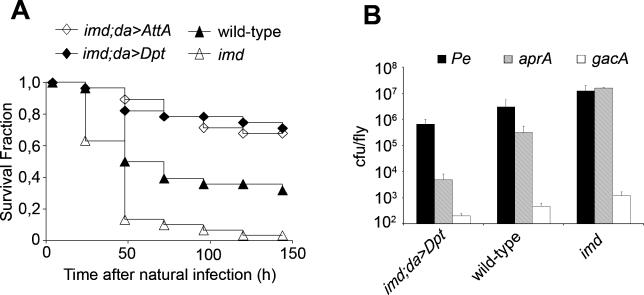
AprA Confers Protection against Diptericin (A) *imd* flies over-expressing *Diptericin* or *Attacin A* show increased resistance against P. entomophila infection. Survival rates were monitored on *imd* mutants, wild-type, and *imd*;*UAS-AMP* flies orally infected with P. entomophila (OD_600_ = 25). The genotypes of the utilized flies are: *imd*: *imd*/*imd*; wild-type: *imd/+*; *da-Gal4/+*; imd; da>AMP: *imd*, *UAS-AMP*/*imd*; *UAS-AMP*/ *da-GAL4*. Log-rank analysis demonstrated a statistically significant difference in survival of *imd*;*UAS-AMP* flies and wild-type flies to P. entomophila oral infection (*p* < 0.01). (B) Persistence of wild-type P. entomophila and *aprA* mutants in *imd* flies over-expressing *Diptericin* under the control of the *da-Gal4* driver. *aprA* mutants persist less than wild-type P. entomophila in lines over-expressing *Diptericin* in contrast to flies mutated in the *imd* locus. The number of cfu per fly represented in each histogram corresponds to the average of six independent experiments (± standard deviation). cfu, colony-forming unit.

We have shown that AprA protects P. entomophila against the Imd-regulated immune response. This immune pathway regulates the expression of several AMP genes, as well as many other immune genes. Previous studies indicated that *Attacin A* and *Diptericin* are the AMP genes most strongly induced in the gut following ingestion of infectious bacteria [7]. It has also been suggested that proteases homologous to AprA in other bacterial species degrade AMPs in vitro, thereby enabling pathogens to withstand the attack of the host immune system [20,21]. We confirmed that P. entomophila AprA rapidly degrades Cecropin A in vitro (unpublished data). To investigate a possible in vivo role of AprA in the protection against AMPs synthesized in the gut, we compared survival to P. entomophila in *imd-*deficient flies over-expressing *Attacin A* or *Diptericin* ubiquitously under the control of the *daughterless Gal4* driver *(daGal4)* using the UAS-Gal4 system (referred to as *imd; da>AMP,* Figure S3B). This strategy allowed us to study the contribution of each antibacterial peptide to the defense against a P. entomophila infection. In a first set of experiments, we compared the survival rate of wild-type, *imd,* and *imd; da>Dpt* or *AttA* flies to natural infection with P. entomophila. Ubiquitous expression of *Attacin A* and *Diptericin* with the *da-Gal4* driver greatly increased the resistance of *imd* mutant flies (Figure 6A). This experiment demonstrates that these AMPs can confer protection against P. entomophila and is in good agreement with a previous study showing that Attacin is the most potent AMP against Gram-negative bacteria [22]. Importantly, it also reveals for the first time a major contribution of Diptericin to host defense, which was not previously detected using systemic injection of microbes [22]. To investigate the in vivo relationship between AMP activity and microbial persistence we next compared the numbers of bacteria of wild-type P. entomophila and *aprA* mutants in *imd* fly lines over-expressing *Diptericin*. Figure 6B shows that the *aprA* mutant persisted less well than wild-type bacteria in *imd* flies over-expressing *Diptericin*. This result clearly indicates an in vivo action of AprA against Diptericin. No effect could be detected when *Attacin A* was over-expressed in agreement with the bacteriostatic mode of action of this AMP (unpublished data). Altogether, our results provide an in vivo demonstration that AprA functions in the protection of P. entomophila against *Drosophila* AMPs.

## Discussion

Pathogens have developed a plethora of strategies that allow them to exploit host resources and resist the attacks of the host immune response. To study how flies deal with infections in a natural context, we investigated the interactions between *Drosophila* and a newly identified entomopathogen, *P. entomophila.* Using a combined genetic approach for both the host and pathogen we reveal the importance of the local immune response in host defense against gastrointestinal infections and provide an in vivo demonstration for the role of a bacterial metalloprotease in protection against AMPs. Our analysis reveals that P. entomophila virulence is multi-factorial, as one might expect, but can be decrypted through genetic analysis of the two interacting partners.

### Local Expression of AMP Genes Plays a Major Role against Food-Borne Infections

The use of GFP reporter transgenes in *Drosophila* has revealed that, in addition to the fat body, AMP genes can be expressed in several barrier epithelia that are in direct contact with microorganisms from the environment [7,23]. The precise relevance of this local immune response in *Drosophila* has not been established to date. The present study demonstrates a key role for the local Imd immune response in the gut against oral infection by bacterial pathogens. The Imd pathway regulates a large number of immune genes including those encoding AMPs; and two peptides, Diptericin and Attacin, are preferentially synthesized following bacterial infection in the digestive tract of *Drosophila* [7]. To explore the potential implication of AMPs in the Imd immune defense against oral infection by *P. entomophila,* we compared survival of flies expressing only a single AMP. Our study reveals that over-expression of either Diptericin or Attacin confers protection against P. entomophila. This observation, combined with our data demonstrating that P. entomophila expresses specific virulence factors in order to resist AMPs, underlines the critical role of local AMP expression against food-borne pathogens.

Previous studies revealed only a modest contribution of Diptericin to resistance against Gram-negative bacteria during systemic infection [22]. In contrast, our data reveal that Diptericin plays an essential role in the defense against Gram-negative bacteria when they are ingested. The antibacterial activity of Diptericin may be enhanced in the gut by pH and other factors such as lysozymes [24]. It is also possible that Diptericin could reach high concentration levels in restricted areas of the *Drosophila* gut such as the proventriculus and thus be more effective against pathogens than in other body compartments. Our study suggests that *Diptericin* expression in the anterior gut provides an efficient early barrier allowing *Drosophila* to rapidly eliminate most ingested bacteria.

Finally, it remains surprising that P. entomophila infection triggers a systemic immune response that has no overt function against the bacteria that remain in the gut lumen. This response could be interpreted as anticipation of possible breaching of the gut barrier. Alternatively, our results suggest the possibility that bacteria such as P. entomophila may subvert insect host defenses by triggering this systemic response. This can be compared with many human pathogens for which activation of an inflammatory response represents a part of their invasive strategy [25].

### AprA Protects P. entomophila from Antibacterial Peptides

In bacteria, nitrogen and carbon sources are frequently provided by enzymatic degradation of extracellular biopolymers by proteases and glycosidases. Proteases, especially metalloproteases, are also known to contribute to virulence in some pathogenic bacteria including *Pseudomonas aeruginosa, S. marcescens,* and *Bacillus thuringiensis* [20,21,26]. Molecular mechanisms for pathogenesis attributed to these proteases include degradation of structural matrices and destruction of proteins involved in host protective functions such as AMPs or complement factors. However, in most cases, attempts to evaluate the role of metalloproteases in virulence have failed to obtain conclusive results with respect to a specific function [20,21].

Thus, our approach focusing on both host and pathogen is the first to clearly demonstrate a key role of the AprA metalloprotease in the protection against AMPs in vivo. This conclusion is based on our observations that (i) *aprA* mutants show attenuated virulence, (ii) *aprA* mutants survive less well than wild-type P. entomophila in *Drosophila* and are more sensitive to Imd-mediated defense, and (iii) AprA provides specific protection in vivo against Diptericin. Altogether, our study reveals that local AMP expression plays an important role in defense against oral pathogens and that entomopathogens such as P. entomophila can counteract this effect by expressing *aprA*. It is interesting to note that *S. marcescens,* another potent oral pathogen of *Drosophila,* also expresses a protease that can degrade AMPs in vitro [12]. However, in the absence of a *Serratia* protease mutant, the in vivo relevance of the protease is not yet established. Taken together, this observation and our findings suggest that proteases may represent a common strategy used by *Drosophila* pathogens to circumvent the potent antimicrobial host defense.

We cannot exclude that AprA degrades other immune effectors or participates directly as a toxic factor by degrading the gut epithelium or the peritrophic matrix. In *B. thuringiensis,* it has been proposed that the protease InhA2 participates in the degradation of the gut, thus facilitating the action of the Cry toxins [27,28]. Alternatively, in *Photorhabdus luminescens,* an AprA homologue, PrtA, has been proposed not to be a virulence factor, but rather to be involved in degrading host tissues following host death in order to promote efficient nutrition and development of its symbiotic nematode host [14,29]. The observation that injection of AprA into the hemocoel is lethal to adult flies supports the hypothesis of additional roles for the protease in bacterial pathogenesis. However, oral ingestion of purified AprA induces only low levels of lethality, indicating that AprA alone is not sufficient to kill the host via this route of entry, and other factors participate in oral toxicity.

### P. entomophila Virulence Is Multi-Factorial

In agreement with previous studies, we show that *aprA* expression in P. entomophila depends on both PrtR and the GacS/GacA system. The GacS/GacA two-component regulatory system is conserved in numerous Gram-negative bacteria and has been shown to regulate a wide variety of cellular functions and virulence factors [30,31]. Our study indicates that it is the master regulator of P. entomophila virulence. In a *gacA* mutant background, AprA synthesis is not restored when a plasmid with the *aprA* locus is expressed in trans (unpublished data), which is in agreement with post-transcriptional regulation of *aprA* by *gacA* via the two small non-coding RNAs RsmY and RsmZ [11]. As opposed to the pleiotropic effects of GacS/GacA, PrtR appears to be a more specific regulator of *aprA* expression in *P. entomophila,* reminiscent of *aprX* regulation in P. fluorescens [32].

Many pseudomonads and other bacteria express proteases similar to AprA but are not able to infect *Drosophila* by oral ingestion. This indicates that AprA is not the sole virulence factor required for persistence in the *Drosophila* gastrointestinal tract. The difference in pathogenicity exhibited by *gacA, prtR,* and *aprA* mutants underlines the complexity of P. entomophila virulence factors. The observation that both *aprA* and *prtR,* but not *gacA,* mutants retained the capacity to trigger a systemic immune response indicates a clear distinction between pathogenicity and immune activation. Our current hypothesis is that systemic immune activation is linked to bacterial persistence in the gut and release of peptidoglycan fragments small enough to cross the gut barrier [33]. Our results indicate that the GacS/GacA two-component system regulates one or several genes that promote bacterial survival in the gut. This hypothesized persistence-promoting factor may have a function similar to the *Erwinia virulence factor (evf)* gene of *E. carotovora Ecc15* that promotes persistence of Gram-negative bacteria in the larval gut [34]. Since *gacA* mutants did not persist in *imd*-deficient mutant hosts, we speculate that this persistence-promoting factor provides general protection against gut intestinal conditions rather than a specific protection against the fly immune response.

Another interesting feature of P. entomophila virulence is the food-uptake cessation which is observed in *prtR* and *aprA* but not in *gacA* mutants. Food-uptake cessation or blockage in insects is induced by several other entomopathogenic bacteria including S. entomophila and *Yersinia pestis,* enabling persistence in the digestive tract of their insect hosts [35,36]. This observation suggests that peristaltic movements of the gut may also play an important role in the elimination of bacteria, and that entomopathogens have developed strategies to abrogate these movements. In *S. entomophila,* it has been shown that genes encoded by a prophage were responsible for the anti-feeding reaction in its natural insect host, the grass grub Costelytra zealandica larvae [35]. Y. pestis is able to multiply in the flea midgut and forms cohesive aggregates. The absence of homologous genes to these factors in P. entomophila indicates that other factors are probably implicated in this bacterium [10]. Determining the cause of this food-uptake cessation and its possible link to the persistence-promoting factor will be essential for the elucidation of the initial events involved in gut colonization. Finally, our study shows that *aprA,* but not *gacA,* mutants of P. entomophila retain a moderate capacity to kill both adult flies and larvae. This confirms the existence of other bacterial virulence factors. The genome of P. entomophila contains several genes encoding putative insecticidal toxins (e.g., Tc toxins, hemolysins, and lipopeptides). Therefore, it remains to be determined whether the strategies developed by P. entomophila to persist in the larval gut and to kill its host involves genes related to those identified in other entomopathogenic bacteria and how the different factors contribute to pathogenesis.

## Materials and Methods

### Insect stocks.

Oregon^R^ (Or^R^) flies were used as a wild-type strain. The *Rel^E20^* and the *Diptericin-lacZ (Dpt-lacZ), Diptericin-GFP (Dpt-GFP), UAS-imd heat shock (hs)-Gal4, caudal (cad)-Gal4,* and *daughterless (da)-Gal4* fly lines have been previously described [37–39]. *Caudal-Gal4* is expressed in the posterior region of the midgut and in the Malpighian tubules [38]. By standard genetic crosses, we generated *imd* flies carrying two copies of an *UAS-AMP* and one copy of *da-Gal4*. Additional information on these fly lines is provided in [22]. The fly lines *UAS-Rel; Rel,* and *caudal (cad)-Gal4/Cyo; Rel* (kindly provided by Won-Jae Lee) were used to produce *Rel* flies expressing *Rel* only in the gut (UAS-*Rel*/*cad-Gal4*; *Rel^E20^*). F1 progeny carrying the *cad-GAL4* driver were transferred to 29 °C at late larval-early pupal stages for optimal GAL4 efficiency. *Drosophila* stocks were maintained at 25 °C. Infected larvae or adults were incubated at 29 °C.

### Bacterial strains, plasmids, and culture media.

P. entomophila and *E. carotovora Ecc15* have been previously described [8,9]. *Ecc15* induces a systemic immune response after oral infection in larvae but not in adults. P. entomophila rif^R^ and *Ecc15* rif^R^ were cultured in LB medium with appropriate antibiotics when required (rifampicin 100 μg/ml and carbenicillin 600 μg/ml) at 30 °C in Luria-Bertani (LB) medium. Escherichia coli BW25142 was used for cloning experiments and was grown at 37 °C in LB medium. *E.coli* SM10λpir was used to replicate plasmids based on the R6K replicon. The R6K-based pKNG101 plasmid was utilized for in vivo allelic replacements as described before, and the pMTL22 vector was applied for general cloning manipulations [40].

### **Construction of the *aprA* knockout mutant and complementation study.**

An *aprA* knockout mutant was constructed by a double crossover of the suicide vector pKNG101 containing an *aprA* fragment inactivated with a tetracycline resistance cassette. A 2,085-bp *aprA* fragment was cloned with *Xho*I into the multi-cloning site of the ampR resistant pMTL22 vector. The *aprA* fragment was inactivated by inserting the 1,402-bp tetracycline resistance cassette in the unique *Acc65*I site. The 3.4k-bp *Xho*I/*Xho*I DNA fragment was then cloned into *Sal*I digested pKNG101 suicide vector. The construct was named pKNG101-A, and transformed into E. coli SM10λpir. This E. coli strain was subsequently conjugated with P. entomophila. Detection of double recombination events was performed as previously described [40]. *AprA* knockout mutants were confirmed by PCR. To complement the *aprA* mutants, the entire *aprA* operon was subcloned from a pBeloBAC derivative containing the region surrounding the *aprA* operon with *EcoR*I/*EcoR*I into pUCP20 [41] cleaved with EcoRI. The plasmid with the incorporated *apr* operon was called pUCP20*-apr*. To specifically inactivate *aprA* gene, a 120-bp fragment containing a part of the promoter region and the initiation codon of the *aprA* gene was cloned in frame with *Nde*I and *Eco*RV into the *Nde*I/ *Eco*RV-digested pUCP20*-apr* vector. In this plasmid construct, 730 bp of the *aprA* gene were deleted. This strategy produces an in frame deletion that should not have polar effects on downstream gene expression. The proteolytic activity was assessed on 5% skim milk agar plates.

### **Infection experiments.**

Natural bacterial infection of larvae: Approximately 200 third instar larvae were placed in a 2-ml tube containing 200 μl of a concentrated bacterial pellet (optical density at 600 nm [OD_600_] = 200) from an overnight culture and 400 μl of crushed banana. The larvae, bacteria, and banana were thoroughly mixed in the microfuge tube, which was closed with a foam plug and incubated at room temperature for 30 min. The mixture was then transferred to a standard corn meal fly medium and incubated at 29 °C. Larvae were collected at different time points for RT-qPCR analysis and bacterial counts. For bacterial counting experiments, larvae were first rinsed in water, dipped in 70% ethanol (three times, 5 s) for external sterilization, and then homogenized and spread onto LB plates containing rifampicin (100 μg/ml).

Larval feeding with concentrated supernatant or pure protease: Approximately 200 third instar larvae were placed in a 2-ml tube containing 200 μl of 60-fold concentrated filtered bacterial culture supernatant and incubated at room temperature for 30 min. The mixture was then transferred to an apple juice medium plate which was sealed with parafilm and incubated at 29 °C. Dead larvae were counted at indicated time points after infection.

Natural bacterial infection of adults: For oral infection, flies were incubated 2 h at 25 °C in an empty vial in order to starve them and then placed in a fly vial with a filter soaked in a food solution. This food solution was obtained by mixing a pellet of an overnight culture of bacteria with a 5% sucrose solution in equal parts. The final bacterial concentration was OD_600_ = 100 except when otherwise mentioned.

Injection of flies: Solution (buffer, culture supernatant, or protease solution) was injected into the thorax of female adults (aged 3–4 d) with a Nanoject apparatus (Drummond, Broomall, Pennsylvania, United States).

### **Protein analysis and AprA purification.**

A concentrated supernatant was prepared by the centrifugation of an overnight bacterial culture. The supernatant was filtered through a 0.45-μm filter. The clarified solution was concentrated 60-fold by using 5-kDa cutoff Centricon membranes (Amicon^TM^, Millipore, Billerica, Massachusetts, United States)

Proteins were analyzed by SDS-PAGE. Culture supernatant fractions were prepared by centrifuging bacterial cultures at 14,000 *g* and 4 °C for 5 min and precipitating supernatant proteins with 10% trichloroacetic acid (TCA) for 30 min on ice. Precipitated proteins were pelleted at 14,000 *g* and 4 °C for 30 min and washed once with 500 μl cold (−20 °C) 80 % acetone, followed by re-suspension in sample buffer and analysis by SDS-PAGE. For AprA purification, P. entomophila was grown in 1 L of LB medium at 29 °C to the late stationary phase of growth (24 h). The culture supernatant was retained following centrifugation of the culture at 8,000 *g* (4 °C) for 30 min. Solid ammonium sulphate was added to the supernatant to a final saturation of 80%, and proteins were precipitated at 4 °C for 2 h with gentle stirring. Precipitated material was collected by centrifugation at 10,000 *g* (4 °C) for 30 min, and the pellets were combined and solubilized in a minimum volume of 50 mM Tris-HCl [pH 8.5], 1 mM EDTA. The resuspended pellet was dialyzed against 1 L of buffer with two buffer changes at 4 °C. Chromatographic procedures were performed on an ÄKTA FPLC (Amersham Biosciences, Little Chalfont, United Kingdom) system. Following a final clarification step of the solubilized supernatant by centrifugation at 10,000 *g* (4 °C) for 30 min and filtering through a 0.45-μm filter, the solubilized proteins were loaded onto a MonoQ HR5/5 column (Amersham-Pharmacia Biotech) that had been equilibrated with 50 mM Tris-HCl [pH 8.5], 1 mM EDTA. The protease activity of the fractions was determined using azocasein (see protease assay). Fractions containing protease activity were found in the flow-through which was subsequently concentrated with an Amicon Ultra 5000 MW cut-off filter and loaded onto a size exclusion chromatography column (Superdex 200 10/300 GL Tricorn, Amersham-Pharmacia Biotech) equilibrated in running buffer (20 mM Tris-HCl, 5 mM CaCl_2_, 150 mM NaCl [pH 8.0]). Fractions were collected at a flow rate of 1 ml/min and assayed for protease activity as described below.

### Protease assay.

Supernatant samples were assayed for proteolytic activity using azocasein (Sigma, St. Louis, Missouri, United States) as a substrate. Aliquots (50 μl) of samples were added to 200-μl azocasein (5 mg/ml) 50 mM Tris-HCl [pH 8.5], 1 mM EDTA. Fractions were then incubated at 37 °C for 30 min. Non-digested azocasein was precipitated by adding 400 μl of 20% trichloroacetic acid (TCA) to the incubations and centrifuged at 10,000 *g* for 10 min. The supernatants were transferred to plastic cuvettes containing 250 μl of 2M NaOH. The absorbance values of the resulting supernatants were measured at 440 nm. Increased absorbance indicates the presence of proteolytic activity. The blank was obtained by precipitating the substrate plus the sample in TCA without incubation.

### RT-qPCR.

For *Diptericin* mRNA quantification from whole animals, RNA was extracted using RNA TRIzol™. cDNAs were synthesized using SuperScript II (Invitrogen, Carlsbad, California, United States) and RT-qPCR was performed using dsDNA dye SYBR Green I (Roche Diagnostics, Basel, Switzerland). Primer pairs for *Diptericin* (sense, 5′-GCT GCG CAA TCG CTT CTA CT-3′ and antisense 5′-TGG TGG AGT GGG CTT CAT G-3′), and control primers for *rp49* (sense 5′-GAC GCT TCA AGG GAC AGT ATC TG-3′, and antisense 5′-AAA CGC GGT TCT GCA TGA G-3′) were utilized. SYBR Green analysis was performed on a Lightcycler (Roche). The amount of mRNA detected was normalized to control *rp49* mRNA values. We used normalized data to quantify the relative levels of a given mRNA according to cycling threshold analysis (ΔCt). For the Y-axis, we used the value ΔCt *Dipt*/ΔCt *rp49* normalized to control ΔCt *Dipt*/ΔCt *rp49* (100%).

## Supporting Information

Figure S1 The *aprA* Mutant Exhibits Attenuated Virulence towards Adults Survival analysis of *Drosophila* adult flies (*n* = 30) after feeding with wild-type *P. entomophila, gacA, prtR,* and *aprA* mutants; the *aprA* mutant complemented with the wild-type *apr* operon *(pUCP20-apr);* and an *aprA* mutant complemented with the *apr* operon carrying a non-polar mutation in the *aprA* gene *(pUCP20-aprΔaprA).* This experiment was repeated twice and yielded similar results. Log-rank analysis demonstrated a statistically significant difference in survival of flies fed with wild-type P. entomophila and flies fed with the *aprA* mutant (*p* < 0.01).
(1.0 MB TIF)

Figure S2 Over-Expression of Imd in the Gut Protected against P. entomophila whereas Its Activation in the Fat Body Did Not (A) Over-expression of an *UAS-imd* construct with the gut-specific driver *caudal-Gal4* protects flies from an oral infection with *P. entomophila.* No protection was observed when the fat body driver *pumpless-Gal4* was used*.* Log-rank analysis demonstrated a statistically significant difference in survival of wild-type flies and flies over-expressing an *UAS-imd* construct in the gut after oral infection with P. entomophila (*p* < 0.0001).
(B) *Diptericin* expression measured by RT-qPCR in *Drosophila* gut extracted from flies used in (A). A high *Diptericin* expression was observed in the gut of flies carrying both the *UAS-imd* and the *caudal-Gal4* constructs.
(1.2 MB TIF)

Figure S3 Expression of AMP Genes (A) *Diptericin* expression measured by RT-qPCR in *Drosophila* gut extracted from wild-type and *Rel* males following natural infection with P. entomophila. Over-expression of an *UAS-Relish* with the *caudal-Gal4* driver restores the immune inducibility of the *Diptericin* gene. *Diptericin* expression was normalized to *rp49* mRNA. 100% value corresponds to the level of *Diptericin* mRNA obtained after infection of wild-type flies with P. entomophila. *rp49*: *ribosomal protein 49*.
(B) The quantification of AMP gene expression shows that *imd* flies carrying two *UAS-AMP* insertions and *da-Gal4* constitutively express the *UAS-AMP* fusion at a high level. *AttA* (left) and *Dpt* (right) were expressed at 50% and 35% of the level observed in 6 h-bacterial challenged (by injection) wild-type flies, respectively. AMP gene expression was monitored by RT-qPCR. The levels of AMP gene expression were normalized by the corresponding values of the *rp49* signal. *AttA, Attacin A; Dpt, Diptericin.*
(1.8 MB TIF)

### Accession Numbers

The FlyBase (http://flybase.bio.indiana.edu) accession numbers for the *Drosophila* strains produced include *Attacin A* (CG10146), *Caudal* (CG1759), *Diptericin* (CG12763), *Imd* (CG5576), *Pumpless* (CG7758), and *Relish* (CG11992).

Accession numbers for the bacterial genes are *Erwinia carotovora evf* (AY167732), *P. entomophila aprA, aprD, aprE, aprF, aprI, gacA,* and *prtR* (CT573326).

## Abbreviations

AMP: antimicrobial peptide
GFP: green fluorescent protein
Imd: immune deficiency
OD_600_: optical density at 600 nm
*Rel*: *Relish*
RT-qPCR: real time quantitative PCR
